# Expression levels of immune markers in *Actinobacillus pleuropneumoniae *infected pigs and their relation to breed and clinical symptoms

**DOI:** 10.1186/1746-6148-5-13

**Published:** 2009-04-21

**Authors:** Laurentiu Benga, Doris Hoeltig, Thomas Rehm, Hermann-Josef Rothkoetter, Reinhard Pabst, Peter Valentin-Weigand, FUGATO-consortium IRAS

**Affiliations:** 1 Institute of Functional and Applied Anatomy, Hannover Medical School, Hannover, Germany; 2 Clinic for Swine and Small Ruminants, Department of Infectious Diseases, University of Veterinary Medicine Hannover, Hannover, Germany; 3 Institute for Microbiology, Department of Infectious Diseases, University of Veterinary Medicine Hannover, Hannover, Germany; 4 Institute of Anatomy, Otto-von-Guericke University Magdeburg, Magdeburg, Germany

## Abstract

**Background:**

In pigs little is known about the role of innate immune defence in bacterial infections of the respiratory tract, despite their major role in pig production. In the present study we characterized and compared *in vitro *and *in vivo *activation of immune markers of different pig breeds 7 days before, and 4 and 21 days after an experimental aerosol infection with *Actinobacillus (A.) pleuropneumoniae*.

**Results:**

*In vitro *stimulation of bronchoalveolar lavage fluid (BALF) and blood leukocytes with *A. pleuropneumoniae, Streptococcus suis*, PMA and LPS led to production of different amounts of H_2_O_2_, NO and TNF-α, depending on the stimulus, individual, breed and time of infection. Generally, significant responses to *in vitro *stimulation were observed only in blood leukocytes, whereas the alveolar macrophages showed a high basal activation. In addition, the production of haptoglobin and cytokines (TNF-α, IFN-γ and IL-10) *in vivo *was measured in plasma and BALF. Plasma haptoglobin levels mirrored the clinical manifestations at 4 days post-infection. In plasma and BALF TNF-α could not be detected, whereas variable levels of IFN-γ were found at pre- and post-infection times. IL-10 was found in some plasma but in none of the BALF samples. The different expression levels in individuals within the breeds correlated for some markers with the severity of clinical manifestations, e.g. H_2_O_2, _plasma haptoglobin and BALF IFN-γ for German Landrace pigs.

**Conclusion:**

Our findings revealed differences in the activation of the immune markers with respect to infection time, individuals and breeds. Moreover, results showed different correlation grades between the immune markers produced *in vitro *or *in vivo *and the clinical manifestations. Further analyses will have to show whether these markers may serve as correlates of protection against porcine respiratory infections.

## Background

Several respiratory tract pathogens, such as *Mycoplasma hyopneumoniae, Haemophilus parasuis, Bordetella bronchiseptica, Pasteurella multocida*, and *A. pleuropneumoniae*, are the cause of serious losses in pig production. Porcine *Actinobacillus *Pleuropneumonia is a highly contagious, fibrinous, haemorrhagic and necrotizing pneumonia leading to high mortality in acutely infected pigs and persistent lung lesions in chronically infected pigs [1,2].

Following respiratory bacterial infection the host reacts with defence mechanisms of the innate immune system, such as pathogen recognition, phagocytosis, respiratory burst, and production of nitric oxide as well as of cytokines [3-5].

Additionally, non-specific markers for infection and inflammation, such as the acute phase proteins, are induced by the host [6]. The respiratory burst represents the main mechanism of killing intracellular pathogens, via the reactive oxygen intermediates such as hydrogen peroxide (H_2_O_2_) [4]. Furthermore, phagocytes are activated by bacterial infection to produce pro-inflammatory cytokines (i.e. TNF-α) and nitrite compounds such as NO, which act together with products of the respiratory burst leading to an effective antimicrobial response [3,7]. The activation of the immune system in response to infection can lead to an increased concentration of cytokines and acute phase proteins in pig serum or on the mucosa, making them useful as possible infection markers [8]. Markers belonging to these response pathways have been shown to be involved in the antibacterial immune response in pigs and other species [9-14]. The activation of such immune mechanisms may generally define the defence phenotype of an individual, which is probably dictated by a certain genetic disposition. These aspects might possibly influence the different susceptibility/resistance of different individuals/breeds. This is of specific interest in animal production, because selection and breeding of the resistant phenotypes can be used as a means to fight infectious diseases. Recently, studies aimed at breeding based on genetic selection of pigs resistant to different pathological agents have been performed [15,16].

The aim of the present investigation was to study the activation of selected immune markers in different porcine genetic backgrounds and also to define their suitability as markers for resistance or susceptibility, using an experimental aerosol infection with *A. pleuropneumoniae *as a model. Blood and bronchoalveolar lavage fluid (BALF) leukocytes were harvested from four different pig breeds at 7 days pre-infection and 4 and 21 days post-infection and their activation was studied by measuring the production of H_2_O_2_, NO and TNF-α after stimulation with *A. pleuropneumoniae, S. suis*, lipopolysaccharide (LPS) and phorbol miristate acetate (PMA). In addition, the concentration of cytokines and haptoglobin produced *in vivo *was measured in plasma and BALF at pre- and post-infection. In another study (Hoeltig et al.,) [17] we observed that different pig breeds display different resistance/susceptibility to an experimental aerosol infection with *A. pleuropneumoniae*. Based on clinical scores as described by Hoeltig et al., we studied the activation of the immune markers in response to *A. pleuropneumoniae *infection and compared animals of the four breeds with respect to animal-animal variations and between breeds. Moreover, the correlation of the immune and clinical markers was addressed.

## Results

### *In vitro *activation of immune markers in response to *A. pleuropneumoniae *infection

Porcine leukocytes were stimulated *in vitro *with *A. pleuropneumoniae*, and *S. suis *which was included as a gram-positive control. PMA and LPS were used as positive controls, whereas the negative control consisted of non-stimulated cells.

**Figure 1:**
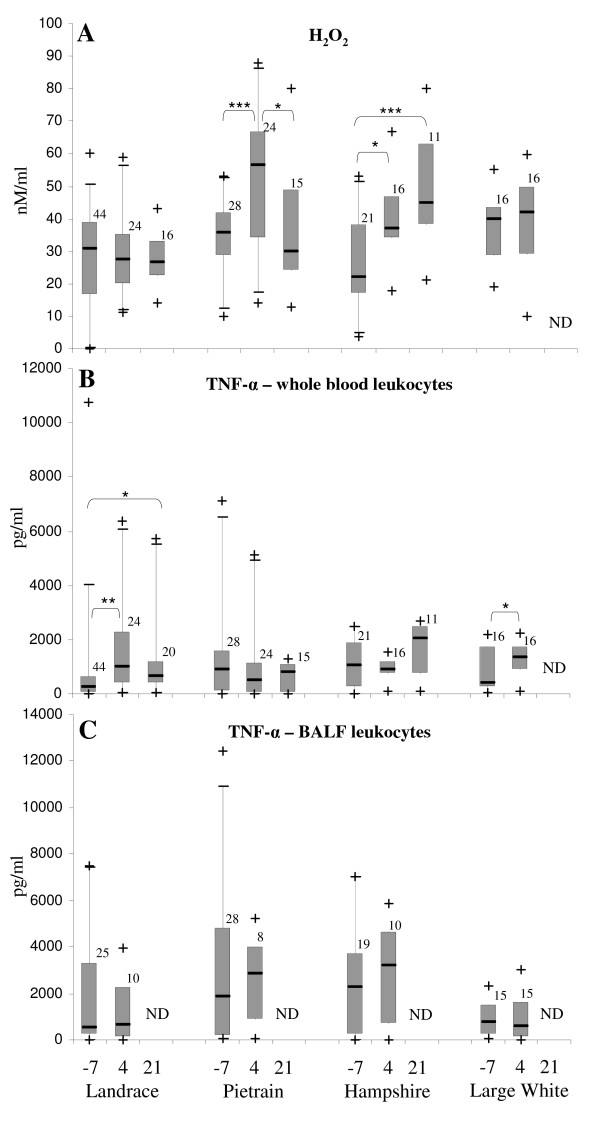
**Kinetics of the activation of immune markers *in vitro *in response to *A. pleuropneumoniae *infection**. H_2_O_2 _(A) and TNF-α (B) production were measured after *in vitro *stimulation of blood leukocytes with PMA (for A) or inactivated *A. pleuropneumoniae *(for B). (C) Basal expression of TNF-α by BALF leukocytes after 6 h cultivation *in vitro*. The expression of the specific markers was recorded as shown on the bottom of the graph for each breed at 7 days before infection (-7) as well as at 4 and 21 days after infection. The box represents the 50% between 25% and 75% quartiles. The line inside the box indicates the median. The top and bottom lines denote the 5 and 95 percentile, whereas the black crosses denote minimum and maximum values. The numbers on the top of the boxes indicate the number of samples examined. ND – not determined; * p < 0.05; ** p < 0.01; *** p < 0.001.

The blood leukocytes efficiently produced H_2_O_2 _in response to PMA stimulation, whereas the stimulation by live *A. pleuropneumoniae *or *S. suis *was slightly above the basal activation of non-stimulated cells (data not shown). Taking this into account, the PMA activation was further used to quantify the respiratory burst of the immune cells. Overall there was a high variation among the individual pigs tested at all time points. For example, at 4 days post-infection, the blood leukocytes of German Landrace pigs produced a mean of 29 nM/ml H_2_O_2 _with a range of 11 to 59 nM/ml. The blood leukocytes harvested at 7 days pre-infection showed no significant change in H_2_O_2 _production in comparison to the cells harvested at post-infection for the breeds German Landrace and Large White (Fig. 1A). In contrast to these breeds, the amount of H_2_O_2 _produced by cells harvested at 4 days post-infection from Pietrain and Hampshire pigs increased significantly in comparison with pre-infection. The production of H_2_O_2 _increased further at 21 days post-infection in comparison with day 4 for Hampshire cells but decreased to values similar to those from pre-infection for Pietrain (Fig. 1A). Pre-infection blood leukocytes from German Landrace, Pietrain and Hampshire pigs produced similar amounts of PMA-induced H_2_O_2_, whereas the cells harvested from Large White pigs produced significantly more H_2_O_2 _than those from German Landrace and Hampshire. Four days post-infection leukocytes from Pietrain, Hampshire and Large White pigs were able to produce significantly more H_2_O_2 _in comparison with the German Landrace cells, whereas at 21 days post-infection, Hampshire cells produced more H_2_O_2 _than the other two breeds (Fig. 1A and Additional file 1).

TNF-α production by blood leukocytes was stimulated in a similar pattern and amplitude by LPS and inactivated *A. pleuropneumoniae*, whereas stimulation with *S. suis *was lower and showed a different pattern (data not shown). Live bacteria proved to be toxic for the cells over the 6 h stimulation time. For this reason, kinetic studies of TNF-α production were carried out following stimulation with inactivated *A. pleuropneumoniae*. Levels of TNF-α in the cell supernatants varied among the individuals and increased significantly from pre- to both post-infection times when cell samples from German Landrace pigs were used (Fig. 1B). Similar to German Landrace pigs, the TNF-α production by blood leukocytes from the Large White pigs increased significantly from pre- to post-infection, whereas the expression of this marker remained constant independent of the infection time when Pietrain and Hampshire cells were used. The amounts of TNF-α produced by cells from Pietrain and Hampshire pigs were significantly higher than those produced by German Landrace cells at pre-infection. At 4 days post-infection German Landrace cells produced significantly more TNF-α than Pietrain cells and Large White cells more than Pietrain and Hampshire cells. At 21 days post-infection the cells harvested from Hampshire pigs displayed significantly elevated levels of TNF-α in comparison with Pietrain cells (Fig. 1B and Additional file 1).

NO production by blood leukocytes was stimulated only by live but not inactivated *A. pleuropneumoniae *or LPS, whereas live *S. suis *induced very low NO amounts of about 3–5 μM/ml (details not shown). Kinetic studies of NO production were carried out after stimulation with a live non-cytotoxic *A. pleuropneumoniae *Apx mutant, because the wild type *A. pleuropneumoniae *proved to be toxic for the cells over the 6 h stimulation period. The NO production varied among the individuals tested (range: 27–101 μM/ml for German Landrace pigs at 4 days post-infection) and did not show any significant differences among the cells harvested at pre- and 4 days post-infection in samples from German Landrace and Large White pigs (data not shown). The NO levels produced by cells from Pietrain and Hampshire pigs decreased significantly at 4 days post-infection in comparison with pre-infection. However, the amounts of NO elicited at all time points were similar for all breeds.

In contrast to blood leukocytes, non-stimulated BALF leukocytes showed a very high basal activation when stimulated *in vitro*. This activation was similar to that of the cells stimulated with PMA, LPS, *S. suis *or *A. pleuropneumoniae *(data not shown). The basal activation decreased after treatment of the cells with 100 μg/ml gentamicin for 2 h before determination of NO production, but remained high when H_2_O_2 _and TNF-α were measured. For this reason, the H_2_O_2 _production by BALF leukocytes was not included in this study, whereas for TNF-α activation the basal production of non-stimulated cells was considered.

Figure 1C shows the production of TNF-α by non-stimulated BALF leukocytes harvested at pre-infection and 4 days post-infection with *A. pleuropneumoniae*. There was a high variation among the individuals tested at both time points, but no significant change in the TNF-α production with respect to infection time for any of the four breeds. By comparing the basal TNF-α production by non-stimulated BALF leukocytes from all breeds, it was found that Hampshire cells produced significantly more TNF-α than the Large White cells at pre- and post-infection and likewise the cells from Pietrain also produced significantly increased levels as compared to those from Large White pigs at 4 days post-infection (Fig. 1C and Additional file 1).

The NO production by BALF leukocytes was elicited only by live but not dead *A. pleuropneumoniae *or LPS, similar to blood leukocytes. In comparison with the other markers, NO expression was more homogenous among individuals (e.g. range 72–104 μM/ml for German Landrace cells at 4 days post-infection; data not shown). There was a significant increase in the amount of NO produced by the Large White cells from pre- to 4 days post-infection, whereas for the cells from Pietrain and German Landrace pigs there was no modification with respect to infection time. Pre-infection, the amount of NO produced by BALF cells was similar for all breeds tested. At 4 days post-infection the amount of NO in the supernatants of the Large White cells was significantly higher than in those from Pietrain and German Landrace cells, whereas the German Landrace cells produced more NO than Pietrain BALF cells (data not shown).

### *In vivo *activation of immune markers in response to *A. pleuropneumoniae *infection

In parallel with the cell stimulation *in vitro*, plasma and BALF samples were tested for the presence of TNF-α, IFN-γ and IL-10, as markers for pro- and anti-inflammatory responses. In addition, plasma concentrations of haptoglobin were measured.

**Figure 2:**
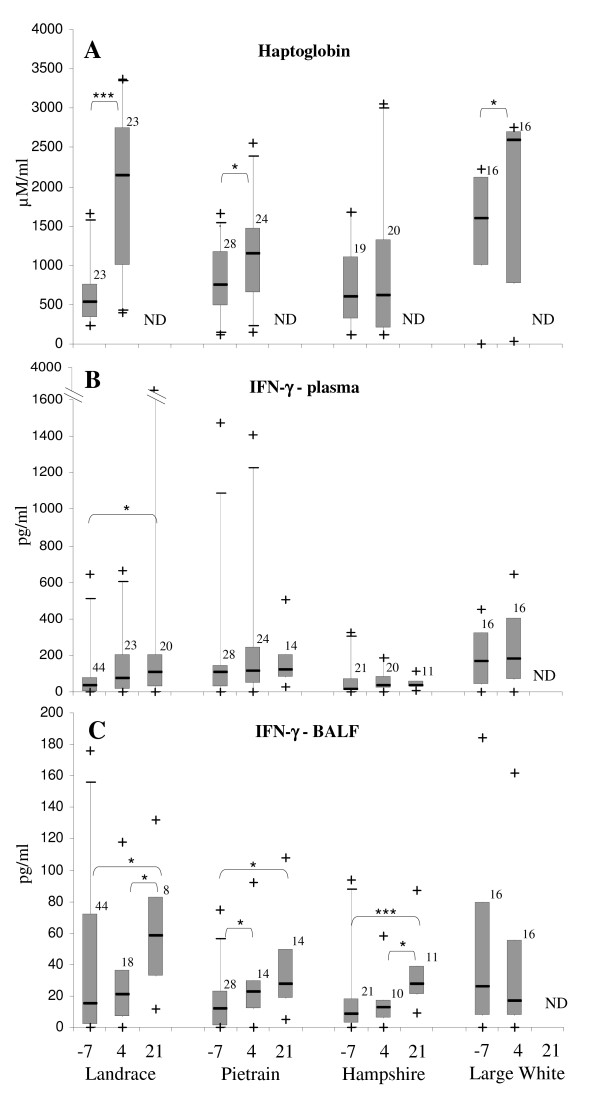
**Kinetics of the activation of immune markers *in vivo *in response to *A. pleuropneumoniae *infection**. The concentrations of haptoglobin in plasma (A) and of IFN-γ in plasma (B) and BALF (C) were measured at 7 days before infection (-7) as well as at 4 and 21 days after infection. The box represents the 50% between 25% and 75% quartiles. The line inside the box indicates the median. The top and bottom lines denote the 5 and 95 percentile, whereas the black crosses denote minimum and maximum values. The numbers on the top of the boxes indicate the number of samples examined. ND – not determined; * p < 0.05; ** p < 0.01; *** p < 0.001.

The haptoglobin concentration in plasma increased significantly in the samples harvested from German Landrace, Pietrain and Large White, but not in those from Hampshire pigs from pre-infection to day 4 post-infection (Fig. 2A). The pre-infection plasma harvested from the Large White breed contained significantly higher levels of haptoglobin than plasma from the other three breeds. The German Landrace and Pietrain samples showed significantly higher values than the plasma samples from the Hampshire pigs at 4 days post-infection (Fig. 2A and Additional file 1).

Preliminary studies revealed that plasma samples tested did not contain detectable levels of TNF-α, at either pre- or post-infection times. For this reason the screening for TNF-α did not include further plasma samples. In contrast, low levels of IFN-γ could be detected in approximately 60% of the plasma samples pre-infection (Fig. 2B). At 4 days post-infection the level of IFN-γ was similar to that at pre-infection, and could be detected in all but three of the German Landrace plasma samples. Twenty-one days post-infection the levels of IFN-γ increased significantly in comparison with pre-infection only in the plasma of German Landrace pigs (Fig. 2B). Plasma from the other three pig breeds also contained low amounts of IFN-γ pre-infection, which did not increase significantly post-infection, in contrast to the German Landrace samples (Fig. 2B). At pre-infection the level of IFN-γ in plasma from Large White pigs was significantly higher than in that from the Hampshire and German Landrace pigs. Four days post-infection the breeds Large White and Pietrain had significantly higher amounts of IFN-γ in plasma than Hampshire. Also at 21 days post-infection, the IFN-γ levels in plasma of Hampshire pigs were significantly lower than those of German Landrace and Pietrain pigs (Fig. 2B and Additional file 1).

IL-10 concentrations higher than 5 pg/ml were detected in 10 of the 44 plasma samples tested at pre-infection in German Landrace. At 4 and 21 days post-infection IL-10 values > 5 pg/ml could be detected in 7 of 23 samples and 11 of 20 samples respectively. The samples of the other three breeds showed similar IL-10 activation patterns (data not shown).

The BALF samples did not contain detectable levels of TNF-α and IL-10, independent of infection time. On the other hand, most of the samples displayed very low levels of IFN-γ at pre-infection and 4 days post-infection. The levels of IFN-γ increased significantly at 21 days post-infection in BALF of German Landrace and Hampshire pigs in comparison with the two other time points (Fig. 2C). For the Pietrain pigs the amounts of IFN-γ also increased significantly from pre-infection to both post-infection time points. Except for the pre-infection time point, at which IFN-γ in BALF from German Landrace was higher than in Pietrain pigs, and from Large White higher than in Hampshire pigs, the IFN-γ amounts were similar for all four breeds post-infection (Fig. 2C and Additional file 1).

### Correlation of the immune and clinical markers

**Figure 3:**
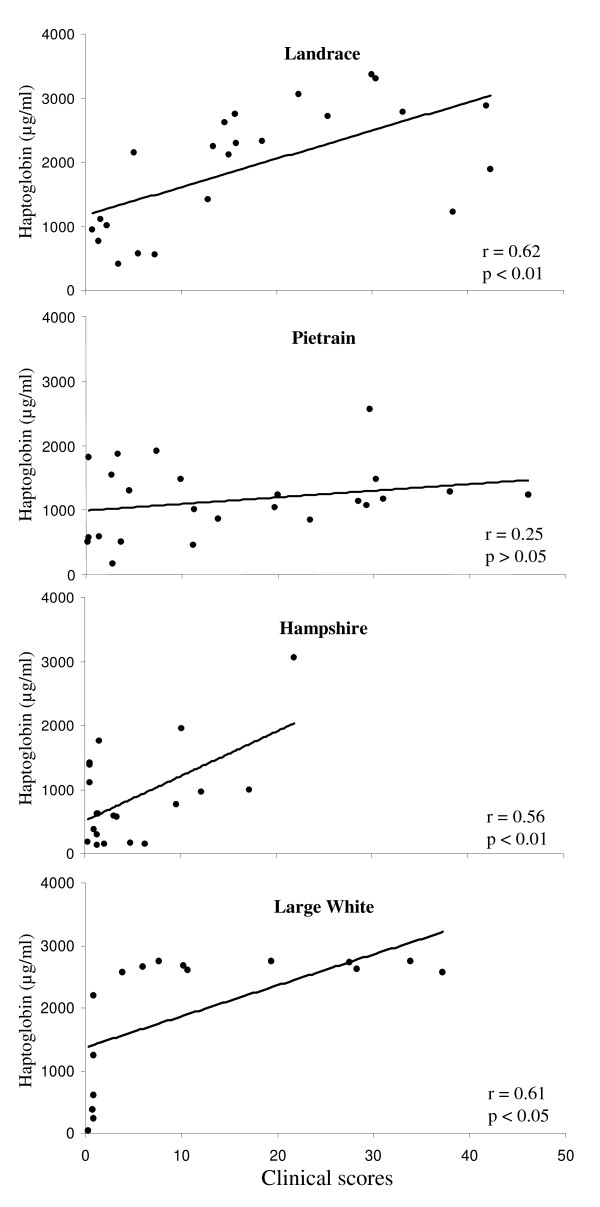
**Linear regression analysis of the clinical scores of the German Landrace, Pietrain, Hampshire and Large White pigs versus plasma concentration of haptoglobin at 4 days after infection**. Each point on the graph represents an individual pig value.

When a linear correlation was performed comparing the level of different markers with the clinical scores of the pigs, different grades of correlation could be established for certain markers and pig breeds. Figure 3 shows the correlation of the clinical scores with the haptoglobin concentration in plasma at 4 days post-infection. The statistically significant correlations for each breed are listed below.

Negative correlations were found at pre-infection between clinical scores and the H_2_O_2 _production (correlation coefficient r = -0.68; p < 0.001), and also between clinical scores and the concentration of IFN-γ in BALF (r = -0.31; p < 0.05) for the German Landrace pigs. Positive correlations were found at 4 days post-infection between clinical scores and haptoglobin in plasma (r = 0.614; p < 0.001) (Fig. 3) for the same breed.

For the Pietrain pigs a negative correlation between the TNF-α production by blood leukocytes *in vitro *and clinical scores was found at pre-infection as well as at 4 and 21 days post-infection (r = -0.45; p < 0.05 at day -7; r = -0.48; p < 0.05 at day 4 and r = -0.61; p < 0.05 at day 21). Positive correlations of the clinical scores with H_2_O_2 _production by blood leukocytes *in vitro *at 4 days after infection (r = 0.61; p < 0.05) as well as between clinical scores and IFN-γ in plasma at 4 days after infection (r = 0.48; p < 0.05) could also be seen for this breed.

For the Hampshire pigs a positive correlation was observed between clinical scores and the concentration of haptoglobin in plasma at 4 days after infection (r = 0.56; p < 0.01) (Fig. 3).

For the Large White pigs a negative correlation was found at pre-infection between the clinical scores and the production of IFN-γ in BALF (r = -0.49; p = 0.05) and in plasma (r = -0.49; p < 0.05). At 4 days post-infection a positive correlation was established between clinical scores and the production of NO by blood leukocytes (r = 0.69; p < 0.01) and also between clinical scores and the plasma concentration of haptoglobin (r = 0.61; p < 0.05) (Fig. 3).

## Discussion

Using a systematic approach we monitored the activation of several immune markers *in vitro *and *in vivo*, at systemic and local respiratory tract level, in response to an experimental aerosol infection with *A. pleuropneumoniae*, and also compared their activation among four different pig breeds.

The differences in susceptibility or resistance observed for different pigs and breeds may also imply differences in the immune system response efficacy. Here, substantial variation of the markers tested was observed between individual pigs and breeds, at both pre- and post-infection times.

Expression of selected immune markers after stimulation of the blood and BALF leukocytes harvested at pre- and post-infection times *in vitro *could define a certain immune status of the animals during the infection and might represent potential correlates for protection. H_2_O_2 _production by blood leukocytes was strongly induced after stimulation with PMA, but not after stimulation with bacteria. However, stimulation with PMA allowed us to measure the activation of the cellular respiratory burst. The cells harvested at pre-infection produced similar levels of H_2_O_2 _for all but the Large White pigs. In contrast, the cells harvested 4 days post-infection produced an increased amount of H_2_O_2 _in comparison with pre-infection for the breeds Pietrain and Hampshire, but not for German Landrace and Large White pigs. Interestingly, this increase corresponded to an increased resistance to infection, whereas for the most susceptible breed, the German Landrace, the amount of H_2_O_2 _was not different between pre- and post-infection. Moreover, the amount of H_2_O_2 _produced by the cells of Hampshire pigs, the most resistant breed, increased after 21 days post-infection in comparison with day 4.

Most interestingly, a negative correlation (p < 0.001) was found between the clinical manifestation and the respiratory burst activation of the blood cells at pre-infection for the German Landrace pigs. The animals that produced more H_2_O_2 _had lower clinical scores, suggesting that the respiratory burst activation might play an essential role in the first line of defence against *A. pleuropneumoniae*. By contrast, after infection the high activation of the respiratory burst correlated positively with the clinical manifestations for the Pietrain pigs. Equilibrium between activation and repression of the respiratory burst seems to be important in the outcome of the disease.

In accordance with published data [14], immune cells stimulated *in vitro *with *A. pleuropneumoniae *or bacterial components were able to induce high amounts of TNF-α. In our case the stimulation pattern was similar after stimulation with LPS and inactivated *A. pleuropneumoniae*, but differed in comparison to stimulation with *S. suis*, suggesting that the activation occurred not specifically by LPS, which is a major part of *A. pleuropneumoniae *inactivated preparations [13]. In two of the three most susceptible breeds (German Landrace and Large White) the ability of blood leukocytes to produce TNF-α *in vitro *increased from pre- to post-infection (see fig. 1B). Although no differences in the intensity of TNF-α production were found between the cells harvested from pre- to post-infection for the Pietrain pigs, a negative correlation of this marker with the clinical scores at pre-infection and at 4 and 21 days post-infection was observed for this breed. This suggests that the capacity to initiate a systemic inflammatory response might play a role in the outcome of infection.

NO production by porcine immune cells did not occur after stimulation with LPS, in correlation with a previous study [18], or inactivated *A. pleuropneumoniae*. Interestingly, it was induced by live *A. pleuropneumoniae*, independent of toxin production, as shown after stimulation with an Apx mutant, suggesting that active protein production might be involved in this process. Although the level of NO produced by the blood cells *in vitro *did not increase from pre- to post-infection, a direct correlation was seen between the clinical score and production of NO at 4 days after infection for the Large White pigs.

Overall, blood leukocytes responded more specifically to stimuli in comparison to BALF leukocytes, which expressed a high basal activation. The basal production of TNF-α by BALF leukocytes remained constant for all four breeds from pre- to post-infection and showed no correlation with the clinical scores at any of the infection time points. The production of NO by BALF cells increased from pre-infection to 4 days post-infection for the Large White pigs. This increase might also play a role *in vivo*, since *in situ *hybridisation studies following a natural infection with *A. pleuropneumoniae *showed positive signals for NOS2 and TNF-α in alveolar macrophages [10]. Moreover, the inducible nitric oxide synthase was found in BALF together with other antimicrobial substances, which in part are present in increased concentrations during infection [19].

Acute phase proteins have been extensively used as markers for infectious and non-infectious disorders in humans [20], and they are attracting increasing attention in veterinary medicine [21]. In our study the haptoglobin concentration in plasma at 4 days post-infection mirrored the clinical manifestations. The most susceptible breeds to infection, German Landrace, Pietrain and Large White displayed a significant increase in plasma haptoglobin concentration from pre-infection to day 4 post-infection, though for some of the Large White pigs basal levels of haptoglobin were noticed at pre-infection, suggesting that subclinical manifestations might have occurred for these pigs. The plasma amounts of haptoglobin did not increase significantly for the breed most resistant to infection (Hampshire), though singular pigs displayed elevated haptoglobin levels. Correlation studies performed for each individual revealed a positive correlation between plasma haptoglobin and clinical scores for the breeds German Landrace, Large White and Hampshire. This study confirmed the sensitivity of haptoglobin as infection indicator following bacterial infections and is in agreement with other studies that showed an increase in the porcine acute phase proteins including haptoglobin, following bacterial infections [8,21,22]. Recently, it has been documented immunohistologically that in acute and chronic bronchopneumonia in pigs haptoglobin was found in airway epithelial cells and immigrated leukocytes but not in alveolar epithelial cells [23].

The production of cytokines *in vivo *by pigs in response to experimental infections or as model studies for human medicine has been widely studied over recent years [8,14,24-30]. However, the presence of some cytokines in porcine serum and BALF is controversially discussed. In the present study we determined TNF-α, IFN-γ and IL-10 in plasma and BALF. We were not able to detect any TNF-α in porcine plasma and BALF before and after infection. This correlates with other reports [24-26], with the exception of one study, in which a different technique was used [14]. Although *in situ *hybridization of lungs from pigs naturally or experimentally infected with *A. pleuropneumoniae *showed positive signals for TNF-α [9,24], this does not seem to correlate with the measurable systemic TNF-α.

On the other hand, it was possible to detect different amounts of IFN-γ in plasma and BALF. In a previous study basal levels of IFN-γ in porcine serum were measured [24], but they did not show any correlation with clinical findings. However, in a similar infection study no detectable serum levels of IFN-γ were measured [26]. In our study, the amount of IFN-γ in plasma increased significantly at 21 days post-infection for the German Landrace pigs, in correlation with the highest susceptibility to infection, whereas the plasma samples from Hampshire pigs, the most resistant to infection, did not show any increase and the amounts of IFN-γ were lower than those from German Landrace, Pietrain and Large White pigs. Interestingly, a positive correlation was observed between the amount of IFN-γ in plasma at 4 days post-infection and the clinical scores for Pietrain and Large White breeds.

BALF levels of IFN-γ increased significantly from pre-infection to 21 days post-infection for all pig breeds tested, suggesting that a cellular immune response occurs locally a long time period after infection. This increase at 21 days post-infection correlated with an increase of the lymphocyte population in the BALF (data not shown), an important population of immune cells for the respiratory tract of pigs [31]. Similar to IFN-γ, neutrophil products such as the chathelicidin PR-39 increased in BALF but not in serum at 21 days after a similar experimental *A. pleuropneumoniae *infection [32], probably in correlation with an increase in the neutrophil BALF population.

In the present study, very low amounts of IL-10 were measured in plasma but not in BALF of singular individuals. This is in accordance with a previous study [29], but in contrast to another [28]. The number of samples which contained very low levels of IL-10 increased after infection, suggesting that an activation of T helper 2 cells leading to antibody-mediated immunity might occur [33].

## Conclusion

Taken together, our findings indicate that activation of certain immune markers *in vitro *or *in vivo *differs among pig breeding lines and partially correlates with the clinical outcome of the disease. It seems that the breeding line-specific susceptibility to *A. pleuropneumoniae *infection is related to different immune phenotypes, possibly caused by different genetic backgrounds. Further genetic analyses will show whether these parameters can serve as biomarkers of protection against porcine respiratory infections in the future.

## Methods

### Bacterial strains and media

*A. pleuropneumoniae *serotype 7 clinical isolate AP 76 [34] was used for challenge experiments in pigs. Strain AP 76 and its ApxIIa toxin-deficient mutant AP 76 Δ1 [34] were used for cell stimulation *in vitro *either as live or as irradiated inactivated (2500 Gy) bacteria. For challenge experiments strain AP 76 was cultured and prepared as described previously [35]. For the cell stimulation experiments strain AP 76 and its ApxII-a toxin-deficient mutant were cultured over-night at 37°C in PPLO medium (Difco GmbH, Augsburg, Germany) with supplements as previously described [35]. *Streptococcus suis *serotype 2 strain 10 [36] was used as a gram-positive control and cultured as described previously [37].

### Clinical study

Seven week old piglets (German Landrace (n = 44), Pietrain (n = 28), Hampshire (n = 21), and Large White (n = 16)) were infected with *A. pleuropneumoniae *by aerosol infection as described previously [36,38]. Subsequently, the clinical scores, based on several parameters, were recorded as described previously [17,38]. The animal experiments were performed in accordance with the principles outlined by the European Convention for the Protection of Vertebrate Animals used for Experimental and Other Scientific purposes (European Treaty Series, nos. 123  and 170 ; approval number: 33-42502-05/941)

### Blood and BALF samples

All samples (blood and BALF cells, plasma and BALF) were collected at 7 days before and 4 and 21 days after aerosol infection with AP 76, as described previously [39]. Blood and BALF cells were isolated from heparinised blood and BALF respectively, and used immediately. The corresponding plasma and BALF samples were kept at -80°C.

### Isolation and stimulation of primary porcine immune cells

Blood leukocytes were isolated from freshly collected heparinised blood by standard procedures. Briefly, erythrocytes were eliminated by hypotonic lysis, then the remaining leukocytes were suspended in RPMI (Gibco-Invitrogen, Karlsruhe, Germany), supplemented with 10% fetal calf serum (FCS, Gibco-Invitrogen) and 5 mM glutamine to a concentration of 3 × 10^6 ^cells/ml.

BALF leukocytes were harvested directly by centrifugation of BALF (1500 rpm, 5 min.), washed twice in RPMI containing gentamicin (100 μg/ml), and suspended in the same medium as the blood leukocytes (plus gentamicin to decrease the basal activation of the cells) to a final concentration of 2 × 10^6 ^cells/ml.

For stimulation, 100 and 500 μl of each cell suspension were distributed per well in 96 and 24 well plates, respectively, allowed to adhere for 2 h at 37°C and 8% CO_2_, and then washed once with phosphate-buffered saline (PBS) pH 7.4. The adherent cells consisting mainly of monocytes and alveolar macrophages were used for stimulation. To determine the H_2_O_2 _production by porcine leukocytes the stimulation was done in 96 well plates for 1 h in a volume of 100 μl Hanks Balanced Saline Solution (HBSS, Gibco-Invitrogen) containing 0.56 mM phenol red (Sigma, Taufkirchen, Germany) and 20 U/ml horseradish peroxidise (HRPO, Sigma) with PMA (Sigma, 200 nM), or live *A. pleuropneumoniae *and *S. suis *(equivalent of the OD_600 _= 0.02 in PBS) as described before [40]. The cells in the 24 well plates were used to measure their capacity to produce NO and TNF-α in the culture supernatant, after stimulation with 5 μg/ml LPS, live and/or inactivated *A. pleuropneumoniae *and *S. suis *(equivalent of the OD_600 _= 0.02 in PBS) in RPMI, for 6 h at 37°C and 8% CO_2. _Subsequently, the supernatants were harvested and kept at -80°C until the NO and TNF-α were measured.

### Determination of H_2_O_2_, NO and TNF-α production by leukocytes *in vitro*

Measurement of H_2_O_2 _production was performed as previously described [40] with some modifications. The assay is based on the peroxidase-dependent conversion of phenol red by H_2_O_2 _into a compound, which can be determined by measuring absorbance at 600 nm. Briefly, the leukocyte stimulation was performed in 96 well plates, in triplicates, at 37°C and 8% CO_2_, in HBSS containing phenol red and HRPO. The assay was read after 1 h incubation and increasing the pH of the reaction mixture to 12.5, with 10 μl 1 N NaOH, in order to eliminate changes in the absorbance of phenol red due to its behaviour as a pH indicator. The sample concentrations were calculated from a standard curve established using H_2_O_2 _dilutions of known molarities and expressed as nM/ml.

Determination of NO in the cell culture supernatants was performed in duplicates in 96 well plates using the method of Green et al., [41]. Briefly, 100 μl cell culture supernatant were mixed with 50 μl Griess I (1% [w/v] Sulfonilamid; 5% [v/v] H_3_PO_4_) and 50 μl Griess II (0.1% [w/v] N-(1Naphthyl)-Ethylendiamin). After 10 min. at room temperature the absorbance at 550 nm was recorded. Decreasing concentrations of nitrite (100 to 1.56 μM) were used as a reference.

The measurement of TNF-α in the cell culture supernatants was performed using a bioassay described by Bertoni et al., [42] with some modifications. Briefly, 2 × 10^4 ^freshly passaged PK-15 cells were seeded to a 96-well flat-bottom plate in 100 μl DMEM with 7.5% FCS and 5 mM glutamine, and grown over-night at 37°C and 8% CO_2_. Next day the medium was removed and replaced by 50 μl complete medium containing 3 μg/ml actinomycin D (Sigma), which sensitized the cells to TNF-α induced cell death. After incubation for 2 h at 37°C and 8% CO_2_, 50 μl of samples were added to each well. The plates were incubated for an additional 18 h, and then pulsed with 10 μl per well of a solution of 3-(4,5-dimethylthiazol-2-yl)-2,5-diphenyltetrazolium bromide (MTT, Sigma) in distilled water for 2–3 h at 37°C and 8% CO_2_. The reaction was stopped by adding 100 μl lysis buffer (10% [w/v] SDS; 0.01 N HCl). After incubation for 18 h in the dark and at room temperature the OD_550 _was measured. The samples were tested in triplicates. Each plate included a standard curve with medium containing decreasing concentrations (1000 to 1 pg/ml) of recombinant TNF-α (Natutec, Frankfurt/Main, Germany). Wells containing only medium and distilled water were included as controls for maximum viability and maximum lysis, respectively.

### Detection of plasma haptoglobin

Plasma concentrations of haptoglobin were determined using a commercially available kit (Phase™ Range Haptoglobin Assay, Tridelta Development Ltd., Maynooth, Ireland), according to the manufacturer's instructions. The assay is based on the preservation of peroxidase activity of haemoglobin at low pH by combination with the haptoglobin present in the specimen. The peroxidase activity is then developed colorimetrically and measured at 630 nm.

### Determination of TNF-α, IFN-γ and IL-10 concentrations in plasma and BALF

To determine the concentrations of TNF-α, IFN-γ and IL-10 in plasma and BALF the appropriate porcine CytoSet tests from Biosource (Camarillo, CA, USA) were used to develop a sandwich ELISA, as recommended by the manufacturer. Monoclonal antibodies were used as capture antibodies and the biotinylated polyclonal antibodies directed against the cytokines served as detection antibodies. The coating of the plates was done over-night at 4°C in a coating buffer as recommended by the manufacturer. Then the plates were washed, blocked and incubated with BALF (undiluted), plasma samples (diluted 1:1 in an assay buffer provided by the manufacturer), or standard decreasing concentrations of recombinant cytokines, and incubated at room temperature for 1.5–2 h, depending on the cytokine tested. Subsequently, the detection antibodies were added to the plates. After washing, the plates were treated with streptavidin-conjugated HRPO, and after a further washing step the peroxidase activity was measured, using TMB as a substrate. After 30 min the reaction was stopped by adding 1.8 N H_2_SO_4_, and the absorbance at 450 nm was recorded. Cytokine concentrations were calculated based on the standard curve obtained with recombinant standard cytokines, and results were expressed in pg/ml.

### Statistical analysis

A parametric test (ANOVA) was used when the variances were homogeneous, and a non-parametric test (Mann-Whitney) for non-homogeneous variances. For comparison of immune marker expression with clinical scores, linear regression was performed between the X and Y axis using Statistica 7.1. (Statistica for Windows, version 7.1. ).

## Authors' contributions

LB performed stimulation of the cells and the measurement of the immune markers and drafted the manuscript. DH and TR harvested the biological samples and performed the clinical study. HJR, RP and PVW designed and coordinated the study. All authors helped interpret results and have read and approved the final manuscript.

## Supplementary Material

Additional file 1 **Summary of significant differences in the intensity of immune marker expression between the pig breeds**. The data provided show statistically significant differences in the immune marker expression between the pig breeds. Click here for file
