# Supplementary material for: Expression levels of immune markers in Actinobacillus pleuropneumoniae infected pigs and their relation to breed and clinical symptoms
Source: BMC Vet Res. 2009 Apr 21;5:13. doi: 10.1186/1746-6148-5-13 (PMC2678107; doi:10.1186/1746-6148-5-13)
Supplement: Additional file 1 — Summary of significant differences in the intensity of immune marker expression between the pig breeds. The data provided show statistically significant differences in the immune marker expression between the pig breeds. [file 1746-6148-5-13-S1.pdf]

Table I. Summary of significant differences in the intensity of immune marker expression between the pig breeds.

Immune marker tested	Differences between breeds		
	7 day pre-infection	4 days post-infection	21 days post-infection
H_2O_2 (*ex vivo* production by PMA stimulated blood cells)	Large White>Landrace*<br>Large White>Hampshire**	Pietrain>Landrace***<br>Hampshire>Landrace**<br>Large White>Landrace*	Hampshire>Landrace***<br>Hampshire>Pietrain*
TNF-α (*ex vivo* production by *App* stimulated blood cells)	Pietrain>Landrace*<br>Hampshire>Landrace*	Landrace>Pietrain*<br>Large White>Pietrain**<br>Large White>Hampshire*	Hampshire>Pietrain*
TNF-α (*ex vivo* basal production by BALF cells)	Hampshire>Large White*	Pietrain>Large White*<br>Hampshire>Large White*	ND
Haptoglobin (plasma concentration)	Large White>Landrace***<br>Large White>Pietrain***<br>Large White>Hampshire***	Landrace>Hampshire***<br>Pietrain>Hampshire*	ND
IFN-γ (plasma concentration)	Large White>Landrace*<br>Large White>Hampshire*	Large White>Hampshire**<br>Pietrain>Hampshire*	Landrace>Hampshire*<br>Pietrain>Hampshire*
IFN-γ (BALF concentration)	Landrace>Pietrain*<br>Large White>Hampshire***	-	-

*p<0.05; **p<0.01; ***p<0.001; ND – not determined.
